# Wasting syndrome with deep bradycardia as presenting manifestation of long-standing severe male hypogonadotropic hypogonadism: a case series

**DOI:** 10.1186/1472-6823-14-78

**Published:** 2014-09-27

**Authors:** Elena Passeri, Marco Bonomi, Francesco Dangelo, Luca Persani, Sabrina Corbetta

**Affiliations:** 1 Endocrinology and Diabetology Unit, Department of Biomedical Sciences for Health, University of Milan, IRCCS Policlinico San Donato, Via Morandi 30, 20097 San Donato M.se, Milan, Italy; 2 Division of Endocrine and Metabolic Diseases and Laboratory of Endocrine-Metabolic Research, IRCCS Istituto Auxologico Italiano, Piazzale Brescia 20, 20149 Milan, Italy; 3 Division of Internal Medicine, Cernusco sul Naviglio Hospital, Via Uboldo 21, 20063 Cernusco sul Naviglio, Milan, Italy; 4 Division of Endocrine and Metabolic Diseases, IRCCS Istituto Auxologico Italiano, Department of Clinical Science and Community Health, University of Milan, Piazzale Brescia 20, 20149 Milan, Italy

**Keywords:** Hypogonadism, Bradycardia, Hypopituitarism, Heart failure

## Abstract

**Background:**

Physiological functioning of the testes is important for cardiac health besides for virilisation, physical strength, behavior and reproduction; moreover, hypogonadism has been demonstrated as a significant risk marker of increased all-cause and cardiovascular mortality.

**Cases presentation:**

We reported two cases of long-standing hypogonadotropic hypogonadism presenting with wasting, bradycardia and heart failure. The two patients were admitted to emergency department for deep weakness, unresponsive anemia and severe bradycardia, requiring in one case the implanting of a monocameral pace-maker for treatment of heart failure. No previous cardiologic disorders were known and cardiac ischemia was ruled out in both patients. The first patient presented congenital hypogonadotropic hypogonadism combined with mild central hypothyroidism and growth hormone deficiency occurred in the peripubertal age, while the second one was diagnosed with isolated adult-onset severe central hypogonadism. Testosterone deficiency was the main feature in both patients as physical examination revealed clinical stigmata of hypogonadism and testosterone replacement induced a dramatic improvement of general condition. Genetic analysis of genes involved in hypogonadotropic hypogonadism failed to identify alterations.

**Conclusion:**

Long-standing hypogonadism in males can be associated with life threatening body alterations including severe bradycardia and heart failure.

## Background

Hypogonadotropic hypogonadism is defined as a clinical syndrome that results from gonadal failure due to abnormal pituitary gonadotropin levels, resulting from either absent or inadequate hypothalamic gonadotropin releasing hormone (GnRH) secretion or failure of pituitary gonadotropin secretion. The condition may be isolated as well as in association with other pituitary hormone deficiency states. Causes of hypogonadotropic hypogonadism may be acquired, frequently due to structural lesions of the hypothalamic-pituitary region, functional or congenital, as a growing list of genes has been implicated in the molecular pathogenesis of hypogonadotropic hypogonadism.

Clinical presentation of hypogonadotropic hypogonadism depends on the time of onset, as a prepubertal onset lead to delayed sexual development, eunuchoidal proportions, and cryptorchidism as well as the severity of the defect and the presence of associated conditions. Besides infertility and sexual impairment, androgen deficiency is associated with a number of extra-gonadal metabolic changes like decline in energy, depression, reduced muscle and bone mass and increased abdominal fat [1]. Recently, low testosterone levels have been demonstrated as a significant risk marker of increased all-cause and cardiovascular mortality [2], suggesting an emerging role of testosterone on cardiac health in males. Low levels of testosterone have been found to be associated with cardiovascular diseases including heart failure, where hypogonadism could play a role in favoring the onset of some peculiar features like decreased exercise capacity, diminished muscle mass and energy handling and final cachexia [3]. However, isolated hypogonadism is rarely associated with an acute onset with bradycardia and heart failure [4], while hypopituitarism may be life-threatening, especially when adrenocorticotropic hormone deficiency occurs [5], and may present with cardiovascular events like cardiac arrhythmia or congestive heart failure due to dilated cardiomyopathy [6,7].

Here we described two cases of severe long-standing hypogonadotropic hypogonadism associated with acute presentation in an emergency setting of bradycardia, heart failure and wasting syndrome besides the classical hypogonadal phenotype. The first patient presented with multiple pituitary defects as severe hypogonadotropic hypogonadism was associated with mild central hypothyroidism and growth hormone (GH) deficiency, while the second patient was diagnosed as isolated adult-onset severe central hypogonadism. In both patients, androgen deficiency was long-standing and occurred in their very early adulthood.

## Case presentation

### Case 1

A 65 years-old male was admitted to emergency department for heart failure due to severe bradycardia (27 bpm), third degree atrioventricular block, mild hypokinetic cardiomyopathy and anemia [hemoglobin 7.2 g/dl (n.v. 12.0-15.0)]. A monocameral pace-maker was successfully implanted. No previous cardiologic disorders were known. The patients presented lethargic and bed bound from few days, but experienced progressive physical deterioration in the last year. Consanguinity was absent and puberty occurred at the expected age in siblings. At the age of 14 years he was diagnosed for delayed puberty due to isolated hypogonadotropic hypogonadism. The patient was treated with testosterone for few months, then he refused all the therapeutic effort, in agreement with his parents, and spent all his life without testosterone replacement. His cognitive ability was normal, he completed his study and worked as employed till he retired. Indeed, he lived alone with poor socialization. Drug abuse and alcohol consumption were ruled out; he denied the use of any medications. Psychiatric evaluation excluded eating disorders. Physical examination showed feminine appearance characterized by female body fat distribution, high-pitched voice and eunuchoidal body proportions (height 175 cm, arm span 177 cm, BMI 27.2 kg/m^2^). He had minimal body hairs, mild gynecomastia and elephantiasis for chronic lymphedema. Genital examination revealed underdeveloped scrotum, microphallus and bilateral cryptorchidism. Gender dysphoria was absent. Multiple osteoporotic vertebral fractures (T6-T7-T8) were detected by x-ray imaging study. Hormonal assessment was performed two months later, after the stabilization of the cardiac condition, confirming severe hypogonadotropic hypogonadism [testosterone <0.02 ng/mL; follicle-stimulating hormone (FSH) 0.18 mU/mL; luteinizing hormone (LH) <0.1 mU/mL]. Concomitant central hypothyroidism [thyroid-stimulating hormone (TSH) 7.0 mU/L (n.v. 0.4-4), freeT3 2.1 pg/ml (n.v. 2.6-4.4), freeT4 0.65 ng/dl (n.v. 0.9-1.7)], growth hormone (GH) deficiency [peak GH of 1.3 ng/ml after combined stimulation with GHRH 1 μg/kg body weight + arginine 30 g; insulin-like growth factor 1 (IGF1) 25 ng/ml (n.v. 117–252)] and mild hyperprolactinemia (34 ng/ml; n.v. < 20) were diagnosed. Pituitary-adrenal axis function was normal. Pituitary MRI revealed partial empty sella. Testosterone and L-thyroxine replacement was started; six months of therapy induced a mild masculinization, weight gain (+7 kg) and resolution of anemia. Nonetheless, central hypothyroidism and GH deficiency did not resolve (TSH 0.08 mU/L, freeT4 0.83 ng/dl, IGF1 25 ng/ml). The karyotype was 46XY and genetic screening for mutations in the hypogonadotropic hypogonadism genes was negative. In particular, the analysis of the *PROKR2* gene coding sequence, whose allelic variants have recently been described also in cases of hypopituitarism, was negative [8-10].

### Case 2

A 30 years-old man was admitted to emergency department for hypotension, fatigue, anemia and deep bradycardia (32 bpm). He experienced a 19 Kg weight loss in the last year, once-weekly fainting episodes, progressive diffuse myalgia and anxious-depressive disorder preventing the patient to attend minimal daily activity. He was born from consanguineous parents. He suffered from neurosensorial hypoacusia consequent to infantile measles meningitis. Spontaneous puberty occurred at age of 14 years. Since few months he complained low sexual desire and erectile dysfunction, while olfactory was normal. Physical examination revealed BMI 19 kg/m^2^. On genital examination, testes were small (volume 5 ml) and soft in the scrotum, while external genitalia were normal. Cardiac conduction was investigated: Holter-ECG confirmed severe bradycardia with minimal nocturnal heart rate of 28 bpm; any significant pause was recorded. An electrophysiological study failed to demonstrate conduction abnormality. Echocardiography showed normal cardiac function, absence of congenital anomaly; ischemia was ruled out at cardiac stress test. Malabsorption, alcohol and drug consumption and eating disorders were excluded. Two months later the emergency admission, he received an endocrine evaluation that revealed mild hypothyroidism [TSH 5.5 mU/L, freeT3 1.7 pg/ml (n.v. 2.6-4.4), freeT4 0.8 ng/dl (n.v. 0.9-1.7)], low IGF1 levels [107 ng/ml (n.v.117-252)], normal adrenal function and, remarkably, severe hypogonadotropic hypogonadism [testosterone 1.3 ng/ml (n.v. 2.4-10.0), LH 0.6 mU/ml, FSH 1.1 mU/ml]. Pituitary MRI was normal. He promptly started testosterone and L-thyroxine replacement that induced after six months a dramatic improvement of his well-being: the patient experienced a 12 Kg weight gain with improvement of muscle mass and heart rate (56 bpm). Hypothyroidism resolved, L-thyroxine was discontinued and IGF1 levels normalized. Genetic screening for mutations in the candidate genes for hypogonadotropic hypogonadism, namely *FGFR1, PROK2, PROKR2, GNRH1, GNRH2*, was negative.

## Discussion

The occurrence of hypogonadism during early adult life, when testosterone production rate is maximal, has detrimental effects to the whole body, including the heart. Here, we reported two cases experiencing wasting and deep bradycardia, a life-threatening condition, due to long-standing severe testosterone deficiency occurring during their early adulthood. The link between testosterone deficiency and cardiac impairment was confirmed as bradycardia was completely rescued by testosterone replacement in the Case 2, though in the Case 1 this information was unavailable since a monocameral pace-maker was implanted before starting testosterone replacement.

In this setting, the two patients did not complain about sexual dysfunction as general disease state was dominant in Case 2, while psycho-social reasons blunted this aspect in Case 1. Lack of sexual complaints helped to fail the diagnosis of hypogonadism. Testosterone deficiency was the main finding and hypogonadal features were evident when appropriately investigated. Typical gonadal-related features, namely small and soft testis, small size penis, reduced body hair did not catch the attention of physicians during initially evaluation as extra-gonadal features were prominent. In particular, both patients showed severe reduction in skeletal muscle mass and strength, hypoxic condition due to heart failure, in Case1, and severe anemia and bradycardia with hypotension, in Case 2.

In both cases, gonadal failure had a pituitary origin not associated with organic lesions; nonetheless, the screening for mutations of genes involved in hypogonadotrophic hypogonadism was negative.

At the presentation in both patients hypogonadotropic hypogonadism was associated with low IGF1 and low freeT4 levels associated with mildly elevated TSH levels. Thyroid autoimmunity and goiter were absent. On serum samples, the diagnosis of central hypothyroidism is usually suggested by the finding of low freeT4 concentrations associated with low/normal TSH levels. Nevertheless, some central hypothyroid patients have high serum immunoreactive TSH levels but are devoid of full biological activity. In these cases, TSH elevations are similar to those generally found in subclinical or mild primary hypothyroidism and may lead to the misdiagnosis [11]. Nonetheless, differential diagnosis of central hypothyroidism includes non-thyroidal illness syndrome, which occurs in the presence of comorbidities. Patients with non-thyroidal illness syndrome have values of thyroid function testing that largely overlap with those with central hypothyroidism. Considering the outcomes of the two cases, Case 1 was diagnosed with central hypothyroidism, while Case 2 findings were consistent with non-thyroidal illness syndrome due to detrimental hypogonadism. A similar pattern of presentation was observed for GH secretion. Supporting the pituitary origin of the hormonal deficiency in Case 1, mild hyperprolactinemia, usually seen in pituitary defects and secondary to the hypothalamic thyrotropin-releasing hormone (TRH) stimulation, was detected.

Though the effect of central hypothyroidism on cardiac presentation could not be ruled out in these patients, the mild freeT3 reduction and the recovery of euthyroidism in the Case 2 supported a dominant role of long-standing gonadic failure rather than hypothyroidism in determining the clinical presentation. It is worth noting that in the Case 1, GH and TSH deficiencies were likely to become manifest later than hypogonadism as the patient had been previously diagnosed with isolated hypogonadotropic hypogonadism and no growth failure or short stature were detected.

The impact of testosterone on the cardiovascular system is controversial. Nonetheless, testosterone seems to be beneficial to the heart and low levels of testosterone negatively affect cardiovascular system [3]. Moreover, testosterone has been shown to exhibit potential antiarrhythmic properties in the form of decreasing action potential duration and shortened QTc interval [12] and low levels of testosterone in obese men have been suggested to contribute to their arrhythmogenic profile [13].

## Conclusion

Long-standing severe central hypogonadism in males is here reported to be associated with life-threatening body alterations including severe bradycardia. We suggested that unexplained bradycardia or heart failure in an otherwise healthy heart might require a metabolic-endocrine screening comprehensive of pituitary function evaluation.

## Consent

Written informed consent was obtained from both the patients for publication of these Case reports. A copy of the written consent is available for review by the Editor of this journal.

## Competing interest

The authors declare that they have no competing interests.

## Authors’ contributions

SC, EP and FD visited the two patients, performed the hormonal evaluation and made the diagnosis. MB and LP performed the genetic screening for mutations in the hypogonadotropic hypogonadism genes. EP and SC wrote the present manuscript, with the supervision of all the co-authors. All authors read and approved the final manuscript.

## Pre-publication history

The pre-publication history for this paper can be accessed here:

http://www.biomedcentral.com/1472-6823/14/78/prepub
